# Effects of a dual intervention (motor and virtual reality-based cognitive) on cognition in patients with mild cognitive impairment: a single-blind, randomized controlled trial

**DOI:** 10.1186/s12984-024-01422-w

**Published:** 2024-08-01

**Authors:** Jorge Buele, Fátima Avilés-Castillo, Carolina Del-Valle-Soto, José Varela-Aldás, Guillermo Palacios-Navarro

**Affiliations:** 1 Carrera de Ingeniería en Tecnologías de la Información, Facultad de Ingeniería, Industria y Producción, Universidad Indoamérica, Ambato, 180103 Ecuador; 2 Centro de Investigaciones de Ciencias Humanas y de la Educación (CICHE), Universidad Indoamérica, Ambato, 180103 Ecuador; 3 Facultad de Ingeniería, Universidad Panamericana, Álvaro del Portillo 49, Zapopan, Jalisco, 45010 México; 4 Department of Electronic Engineering and Communications, University of Zaragoza, Teruel, Spain; 5 Teruel Polytechnic School of Engineering, University of Zaragoza C/Atarazana, 2, Teruel, 44002 Spain

**Keywords:** Activities of daily living (ADL), Cognitive rehabilitation, Depression, Mild cognitive impairment (MCI), Motor rehabilitation, Virtual reality

## Abstract

**Background:**

The increase in cases of mild cognitive impairment (MCI) underlines the urgency of finding effective methods to slow its progression. Given the limited effectiveness of current pharmacological options to prevent or treat the early stages of this deterioration, non-pharmacological alternatives are especially relevant.

**Objective:**

To assess the effectiveness of a cognitive-motor intervention based on immersive virtual reality (VR) that simulates an activity of daily living (ADL) on cognitive functions and its impact on depression and the ability to perform such activities in patients with MCI.

**Methods:**

Thirty-four older adults (men, women) with MCI were randomized to the experimental group (*n* = 17; 75.41 ± 5.76) or control (*n* = 17; 77.35 ± 6.75) group. Both groups received motor training, through aerobic, balance and resistance activities in group. Subsequently, the experimental group received cognitive training based on VR, while the control group received traditional cognitive training. Cognitive functions, depression, and the ability to perform activities of daily living (ADLs) were assessed using the Spanish versions of the Montreal Cognitive Assessment (MoCA-S), the Short Geriatric Depression Scale (SGDS-S), and the of Instrumental Activities of Daily Living (IADL-S) before and after 6-week intervention (a total of twelve 40-minutes sessions).

**Results:**

Between groups comparison did not reveal significant differences in either cognitive function or geriatric depression. The intragroup effect of cognitive function and geriatric depression was significant in both groups (*p* < 0.001), with large effect sizes. There was no statistically significant improvement in any of the groups when evaluating their performance in ADLs (control, *p* = 0.28; experimental, *p* = 0.46) as expected. The completion rate in the experimental group was higher (82.35%) compared to the control group (70.59%). Likewise, participants in the experimental group reached a higher level of difficulty in the application and needed less time to complete the task at each level.

**Conclusions:**

The application of a dual intervention, through motor training prior to a cognitive task based on Immersive VR was shown to be a beneficial non-pharmacological strategy to improve cognitive functions and reduce depression in patients with MCI. Similarly, the control group benefited from such dual intervention with statistically significant improvements.

**Trial registration:**

ClinicalTrials.gov NCT06313931; https://clinicaltrials.gov/study/NCT06313931.

## Introducction

In recent decades, the increase in human longevity has been notable, with significant transformations in the physical and cognitive domains of aging that demand in-depth analysis [1]. This phenomenon brings significant transformations in the physical and cognitive aspects associated with aging, which requires detailed analysis [2]. Among the cognitive functions affected are perception, attention, language, memory, executive functions, among others [3]. These cognitive skills are essential for people to perform their routine tasks and maintain normal functioning in their environment [4]. The tasks and routines of daily living are called activities of daily living (ADL) and are essential for self-care and independence [5]. There are 11 activities that are considered instrumental (iADL) [6], since they contemplate a greater complexity than the basic activities of daily living (bADL). The decline in iADL performance becomes increasingly notable as MCI emerges [7], which presents a decline in cognitive functions as part of a human pathological state [8, 9].

MCI, also known as mild neurocognitive disorder [10], involves a decline in cognitive abilities that is more pronounced than expected for the individual’s age [11, 12]. Those affected may have difficulties in memory, language, attention and other cognitive functions, but still maintain the ability to carry out daily activities, with less impairment than patients with dementia [13]. However, this review also mentions that cognitive decline is not normal and is a stage prior to the onset of dementia [13], which could generate medical and social repercussions [14]. The risk of MCI and dementia increases in adults over 65 years of age due to exposure to several adverse factors and conditions, including neurodegenerative diseases, dietary changes, and chronic diseases [15, 16]. Furthermore, elements such as gender and ethnicity influence the onset and severity of cognitive impairment [17]. For example, a higher prevalence of diseases such as Alzheimer’s Disease (AD) has been observed in women and in certain ethnic groups [18, 19]. Early identification and intervention are critical to slowing or preventing further cognitive decline [20]. Addressing these disorders from their early stages can significantly improve quality of life, helping people manage the emotional and cognitive challenges that arise, such as frustration, anxiety and depression [21].

Depression in the context of neurodegenerative processes presents complex and significant characteristics. The affective disorders can manifest in various ways in patients with neurodegenerative diseases, exacerbating the clinical and emotional challenges of those who experience them. Depression not only affects patients’ mood, but can also profoundly influence their cognitive ability, daily functioning, and quality of life [22]. Depressive symptoms may include persistent sadness, lack of interest in previously pleasurable activities, changes in appetite and sleep, as well as feelings of worthlessness or excessive guilt. These symptoms can be difficult to distinguish from the emotional and cognitive changes themselves associated with neurodegeneration, further complicating diagnosis and treatment [23]. From a neurobiological perspective, neurodegenerative processes have been observed to affect brain regions involved in mood regulation, such as the hippocampus and prefrontal cortex [24]. The complex interplay between neuronal degeneration and neurotransmitter systems, such as serotonin and dopamine, may contribute to both depressive symptoms and neurodegenerative disease progression. Comprehensive management of depression in patients with neurodegenerative diseases is crucial, not only to improve the patient’s quality of life, but also to optimize cognitive and functional outcomes. Therapeutic strategies that include psychological, pharmacological and rehabilitative interventions may be necessary to adequately address both depressive symptomatology and the specific challenges of the neurodegenerative disease, thus offering a comprehensive and multidisciplinary approach to patient care [25].

Depression is a common comorbidity in people with neurodegenerative disorders, including MCI, often exacerbating cognitive symptoms and affecting overall quality of life. According to the study conducted by Lyketsos et al., depression is one of the neuropsychiatric symptoms that most frequently appear in people diagnosed with AD or MCI [26]. Malhi and Mann [27] mentioned that one out of five individuals experiences an episode of depression during their lifetime, with clinical depression being the third cause of disease burden worldwide according to the World Health Organization (WHO). Additionally, older individuals are more vulnerable to the harmful effects of depression [28, 29]. However, figures reflecting the prevalence of depression in adults present significant variations, influenced by various factors such as the underlying cause, the living environment, the intensity of the disorder, as well as gender and age differences [30–32]. Despite these discrepancies in the figures, it is clear that depression is a common phenomenon in the senile stage, showing a prevalence in depressive disorders [33]. A notable finding is that middle-aged women are almost twice as likely to experience an episode of depression compared to men [34]. This trend continues until advanced age, evidenced by studies such as the one conducted by Curran et al. [28] that indicated that 12% of women present depression as a comorbidity, compared to 8% of men. As Alblooshi et al. indicated, menopause increases vulnerability to depression and anxiety, perhaps through estrogen fluctuations that affect serotonin and gamma-aminobutyric acid (GABA) [35]. Furthermore, decreased estrogen and neurological deterioration after menopause increase women’s vulnerability to developing AD [36].

Given this situation, studies have shown that exposure to cognitively and mentally stimulating activities throughout life protects against cognitive decline, and that engaging in these activities in old age is associated with better cognitive function [37]. Lifestyle factors such as exercise, diet, social participation, having a purpose in life, and cognitive activities can reduce the risk of dementia and improve cognitive function [38, 39]. Advances in computer science and information and communication technologies (ICT) have resulted in increased availability and accessibility of computerized cognitive training [40]. The integration of motor activities in therapeutic interventions has been shown to expand functional capacities in individuals. A systematic review conducted by Zhu et al. [41] included 11 randomized controlled trials that showed a moderate impact of VR-based interventions on improving cognitive and motor function. These improvements included aspects such as attention/execution, memory, global cognition and balance in patients with MCI impairment and dementia.

In aging research, VR emerges as a promising approach for dementia treatment in individuals with cognitive impairment [42]. VR immerses users in virtual environments, delivering visual, auditory, and sensory stimuli via a head-mounted display (HMD) [43]. The recent review by Chan et al. [44] indicated that most interventions utilized traditional desktop computers, followed by touchscreen computers and immersive VR, with the latter accounting for only 5.7% of the total. Despite VR technology not being entirely new, there is a pressing need for further expansion in the literature through new implementations. Gao et al. [45] analyzed six VR interventions combined with traditional rehabilitation, showing significant improvements in general cognition, attention, and mood, although not in global cognition, motor function, and ADL. Recently, cognitive-motor applications have been developed using VR to simulate iADL. A recent review [46] demonstrated that VR-based cognitive-motor interventions activate specific brain areas and improve general cognition, executive function, attention, and memory. While significant progress has been made in understanding VR’s effects on cognition and physical functioning, there remains a notable gap in knowledge regarding its impact on mental health.

Studies such as those carried out by Liao et al. [47], Mrakic-Sposta et al. [48] and Kwan et al. [49] used VR to simulate iADL, examining the combined benefits of VR on physical and cognitive function. However, they did not evaluate the impact VR could have on mental health. Park et al. [50] simulated hygiene and driving activities using VR, highlighting that it could be used to increase motivation, fun and interest, which would improve feelings of depression and lethargy. Doniger et al. [51] conducted an experiment with a virtual supermarket and concluded that VR could not only delay the onset of depression but also standardize and monitor participants’ responses effectively. Liao et al. [52] highlighted the effectiveness of VR in improving ADL performance, although they acknowledged that a significant limitation of their study was the lack of analysis of the impact on depression. These findings highlight the therapeutic potential of VR beyond its physical and cognitive applications, as well as the current limitations in the evaluation of associated variables such as mental health.

All previously presented research analyzing the effects that cognitive and motor VR applications can generate were carried out in high-income countries such as Italy, Taiwan, China, the Republic of Korea and Israel. The literature indicates that cognitive interventions can not only improve cognitive functions but also mitigate depressive symptoms due to the bidirectional relationship between these two domains. However, when analyzing studies that also seek to reduce mental disorders, the trend is similar [53–55]. The concentration of studies in high-income contexts leaves a gap in knowledge about how these interventions could work in regions with different socioeconomic and cultural contexts, such as Latin America (LATAM). This region, composed mostly of upper-middle-income countries, faces unique challenges such as income inequality [56], violence [57], mistreatment of the female gender [58], increased migration and deficiencies in health systems [59, 60].

Increasing life expectancy in the region also implies an increase in the elderly population [61], making the need to develop and adapt effective interventions to improve both cognitive and emotional health even more urgent. The analysis presented by Vigo et al. [62] showed that in South America, investment in mental, neurological and behavioral disorders is lower than the world average. Of the total government budget for the treatment of health conditions, less than 5% is allocated to mental health. This is supported by the literature, where only one VR application was found for detecting Alzheimer’s symptoms but none for cognitive training [63]. In 2021, Brito et al. [64] carried out a sensorimotor intervention based on VR to reduce depression and anxiety in older adults, in Talca, Chile. Trueba et al. [65] presented a VR application, where the elderly were subjected to different nature scenes with the aim of reducing anxiety and depression levels in Quito, Ecuador.

In contrast, our study presents a VR system that fuses physical and cognitive training, simulating a daily life activity. Previous studies have demonstrated that this is the first trial that contrasts the effects of a VR-based cognitive-motor training program with an equivalent pencil and paper program, in a Latin American country. Based on recent studies that used VR to simulate iADL, it was hypothesized that the experimental group will have a significant improvement in cognitive functions post-training. However, uncertainty persists regarding its impact on depressive symptoms and the transfer of skills to daily life. Since these variables are considered secondary in the purpose of the study, we hope to generate novel results that can serve as a starting point for future research focused specifically on depression or on the transfer of skills to daily life, exploring the combination of VR with traditional techniques.

## Materials and methods

### Participants

#### Recruitment

Participants were recruited at a senior community center day care center for older adults in Patate, Ecuador. The center provides social and recreational services for people over 65 years. The center staff invited participants through advertising posters, in-person conversations and through social networks. Subsequently, the research team selected potential participants according to the proposed eligibility criteria.

#### Inclusion criteria

The inclusion criteria were: (1) age equal to or greater than 65 years; (2) attend a day care center without being institutionalized in a geriatric center; (3) maintain physical functionality (absence of any disability); (4) ability to understand the purpose of the study and voluntarily agree by signing the consent form; (5) present MCI; (6) have a Montreal Cognitive Assessment (MoCA) score between 19 and 25 points [66].

#### Exclusion criteria

We excluded patients who presented (1) clinical diagnosis of dementia; (2) probable dementia, with a MoCA score equal to or less than 18 points [66]; (3) neurological disorders, including stroke or traumatic brain injury (TBI) in the past 12 months; (4) history of mental or psychiatric disorders; (5) addiction to medication, drugs or alcohol; (6) difficulties using an HMD and operating the controller equipment; (7) medical conditions that could interfere with effective participation in and completion of the study; (8) visual and/or hearing impairment; (9) communication difficulties.

### Ethics

This preliminary study was designed as a randomized controlled trial (RCT) with parallel groups, conducted at a single center and with single blinding. The objectives and requirements of the study were explained in detail to the participants, who voluntarily gave their consent by signing an informed consent form. Ethical approval was obtained from the Institutional Review Board of the Universidad Tecnólogica Indoamérica, under the code UTI-IIDI-074-2023, approval date May 25, 2023, before starting the study. The trial was registered with ClinicalTrials.gov (identifier number NCT06313931) on March 8th, 2024.

### Randomization

Once baseline data was collected from all participants, the recruitment team evaluated and developed a list of subjects eligible to participate in the study. For their random assignment into groups, they were given a unique number. An independent research assistant then generated a sequence of random numbers (1 for the control group and 2 for the intervention group), using a spreadsheet. Based on this sequence, one author (FA-C) assigned participants to the respective intervention or control groups. This process ensured concealment of the allocation, thus guaranteeing the integrity of the allocation procedure. The person in charge of evaluating the results did not know which group each participant belonged to. However, it was not feasible to maintain anonymity in group assignment, both for those in charge of applying the interventions and for the participants themselves.

### Intervention

#### Study design

**Fig. 1:**
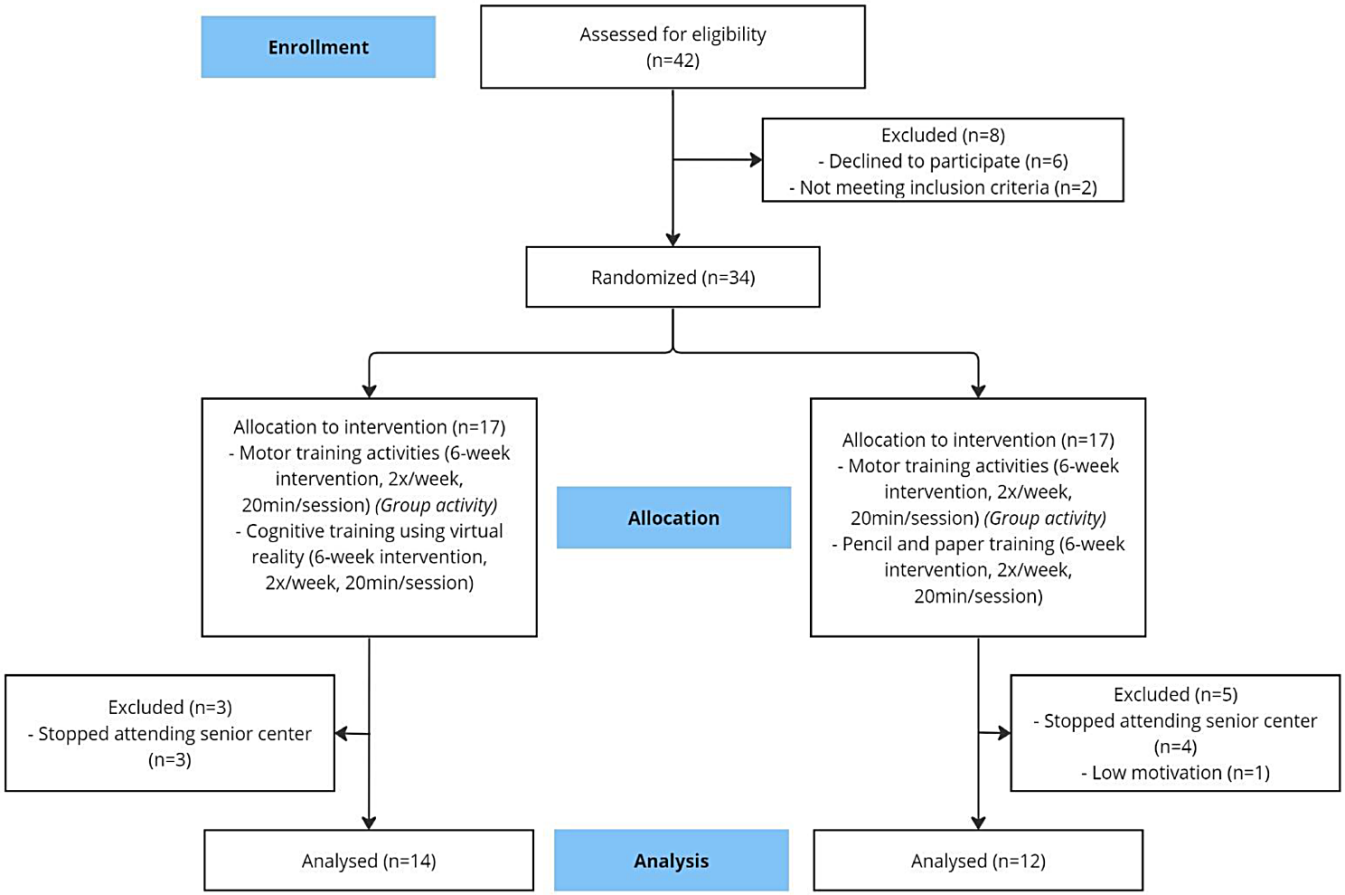
Consort diagram

This study used a pre-post experimental design with a control group to evaluate the impact of the VR-based cognitive-motor training program on cognitive function and depression. Older adults over 65 years who voluntarily expressed interest in participating and met the specified selection criteria were recruited. Two interventions were used: motor training and VR-based cognitive training (*n* = 17) and motor training and traditional cognitive training (*n* = 17), through randomization after taking initial measurements. Previous studies involving VR-based cognitive-motor training used similar samples [47, 50–52]. Five participants were excluded from the study, because they stopped attending the senior center (*n* = 4) and one participant lacked of motivation. In the experimental group three participants stopped attending the senior center. Therefore, the study was completed with a sample of 26 older adults (Fig. 1). Considering that the participants returned to their homes, the usual development of other daily activities was not prohibited. The evaluations were carried out before the start of the motor training program based on VR or pencil and paper (baseline) and after the interventions. Both interventions were conducted twice a week for six weeks, and each session lasted approximately 40 min.

#### Outcome measurements

All training and coaching activities in this study were carried out by researchers with solid knowledge of the subject. To evaluate the effects of the motor-cognitive training programs in both groups, standardized tests were used.

Cognitive function was assessed using the Spanish version of the Montreal Cognitive Assessment (MoCA-S), adapted for use with Spanish-speaking older adults and which was validated in Latin America [67], based on the original MoCA developed by Nasreddine et al. [66]. The evaluation covered attention, verbal fluency, executive functions, short-term memory, visuospatial memory, language, temporal and spatial orientation, and calculations.

Depression was assessed using the Spanish version of the Short Form of Geriatric Depression Scale (SGDS-S), an adaptation designed to assess depression in older adults based on the Geriatric Depression Scale, originally developed by Yesavage et al. [68] and validated in the Ecuadorian population by Erazo et al. [69]. The general emotional state, interest, motivation, perspective, specific concerns and future comparisons were analyzed by means of this scale. The score on the SGDS-S scale ranges from 0 to 15, where a score less than 5 indicates a normal state, whereas a score from 5 to 9 points indicate moderate depression and a higher score indicates moderate to severe depression [69].

Functional ability was assessed using the Spanish version of the iADL scale (IADL-S) was used, an adaptation designed to evaluate the performance of iADL in older adults, validated by Vergara et al. [70]. This test is based on the iADL Scale, originally developed by Lawton and Brody, which is also known as the Philadelphia Geriatric Center-Instrumental Activities Daily Living (PGC-IADL) [71]. Thus, communication, self-care, financial management, mobility, health administration and daily activities were evaluated. Each of the eight items on the scale is coded with a score of 0 (unable or partially capable) or 1 (capable), and responses are summed to obtain a total score. The score range varies from 0 (low function, dependent) to 8 (high function, independent) [71].

#### Experimental group

The training sessions were carried out in a senior community center. These activities complied with the recommendations of the American College of Sports Medicine and the American Heart Association for the older adult [72]. To stimulate the motor system, collective social interaction activities that included conversations between therapists and participants were carried out, promoting non-verbal language, the exchange of thoughts and jokes. Participants were instructed to actively participate in these conversations, use gestures, and share personal anecdotes to enhance interaction. Balance exercises were then performed such as walking in a straight line, placing one foot directly in front of the other, maintaining coordination. Participants were also instructed to focus on a fixed point ahead and maintain an upright posture to ensure stability. In addition, resistance exercises such as squats were performed, with an effort regulated according to the individual capabilities of each person. Participants were reminded to perform squats slowly, keeping their back straight and knees in line with their toes to avoid injury. Finally, a series of whole-body aerobic exercises were performed, such as brisk walking, an accessible exercise for older adults, going up and down stairs, and social dancing individually and in pairs. They were encouraged to maintain a constant rhythm and coordinate their movements with the rhythm of the music during social dancing.

**Fig. 2:**
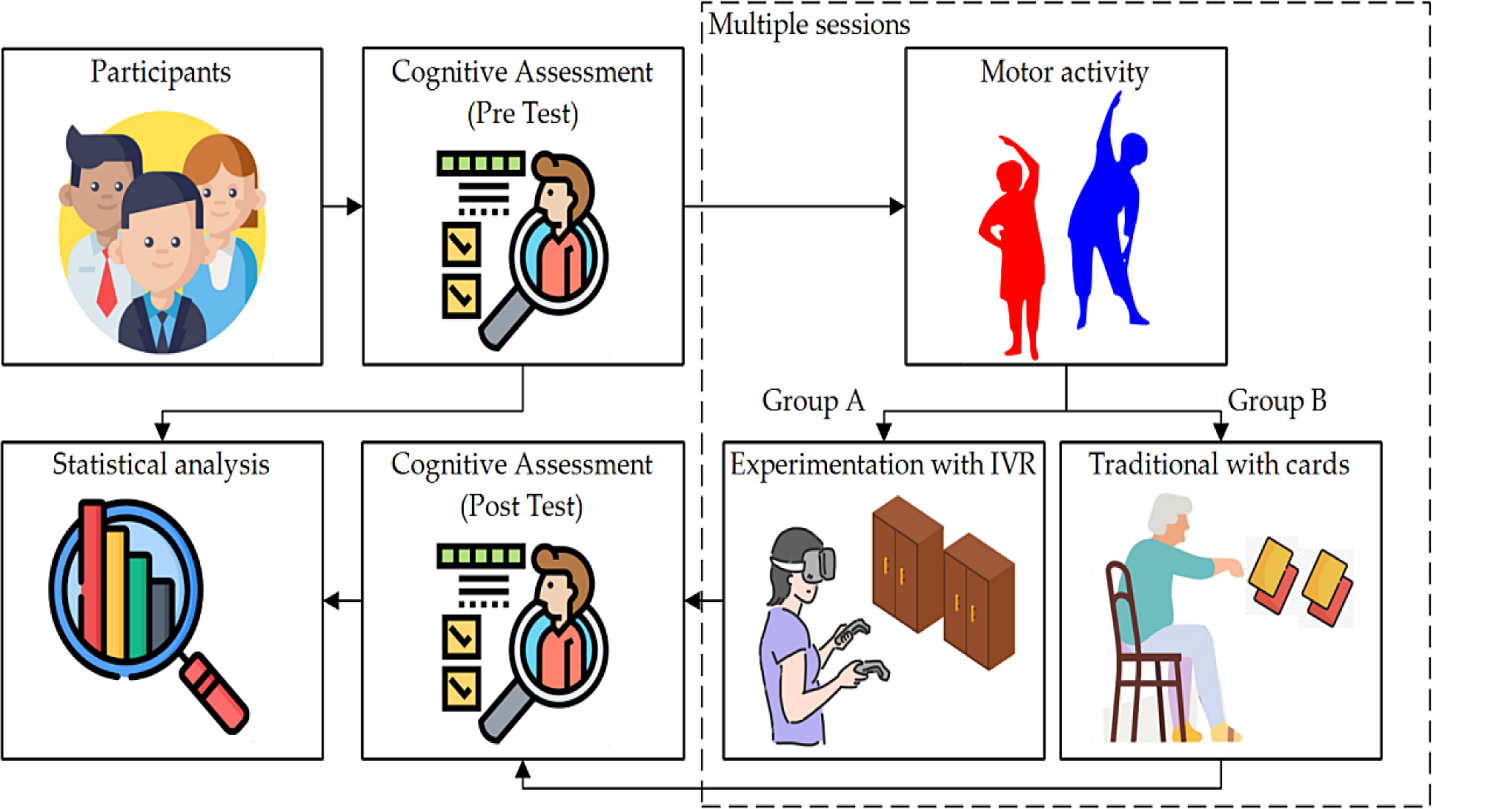
General description of the intervention phases

Subsequently, individual cognitive training was performed using an immersive VR-based system that simulates a task of searching for ingredients in a kitchen cupboard. It includes an application that uses VR to simulate an instrumental activity of daily life (iADL), which was developed with the Unity game engine. The hardware consisted of the Oculus Quest 2 VR goggles and two wireless handheld controllers that allow interaction with the system. Participants were conveniently instructed on the correct use of the VR googles and holding the controllers. They were guided through the VR environment, with clear instructions on how to select and manipulate virtual objects. Since for some of the participants it was their first experience with VR, each session was supervised by a member of the research team, who also solved any technical problems that arose. Figure 2 depicts the intervention phase.

**Fig. 3:**
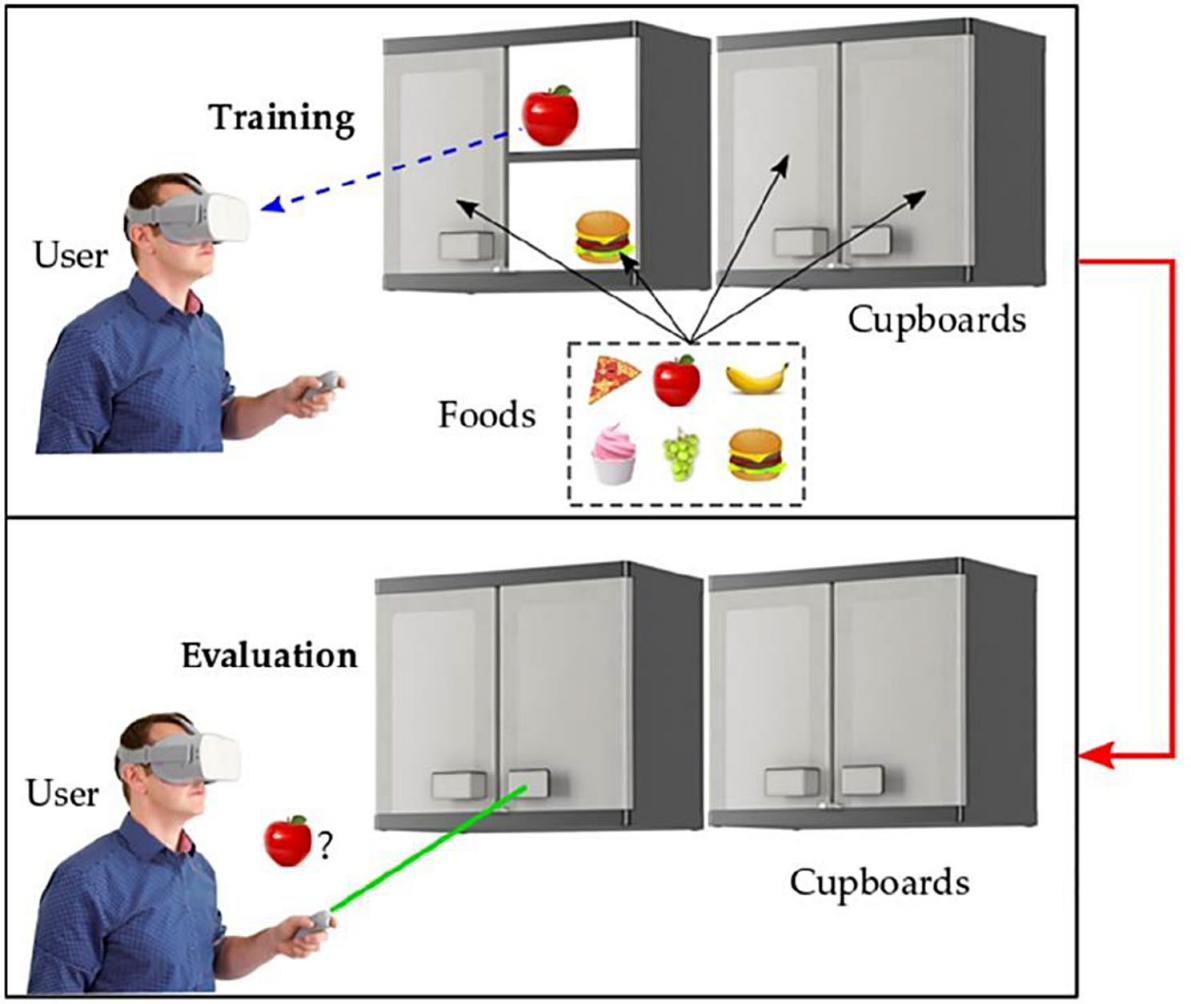
Cupboard task implemented in the VR application

Figure 3 illustrates the dynamics of this serious game. At the beginning, the participant understands the commands to follow and visualizes the ingredients inside every cupboard compartment (registration and coding phase). The items are automatically and randomly placed inside the shelves and the doors are opened individually, with a learning waiting time set at 10 s (storage and consolidation phase). Finally, the participant locates the ingredients randomly requested, selecting both the door and the desired item (retrieval or evocation phase), for which he/she has 10 s per object or an error will be considered and the task will go on by asking a new question. This task tests various cognitive functions such as visuospatial functions (orientation within the virtual environment), attention (registration phase), working memory (storage phase), executive functions (planning of activities), reaction time (responding within the set time).

Given the population under study, it was considered appropriate to start with level 4, which requests memorizing 4 cooking ingredients. Each participant performed the task twice, to prevent possible fatigue or boredom due to monotony. The randomization in the location of the objects allowed the participants to remain active and avoided biases in the performance results. When the participant completed the activity without any error for two consecutive times, the level was increased, i.e., one more item to memorize. The video game has auditory messages that indicate an error or congratulate the user when he/she found a hit. The goal of this design was to ensure that participants had enough time to learn through repeated practice, while at the same time feeling challenged to increase the level, while maintaining their motivation, through a sense of fun.

#### Control group

**Fig. 4:**
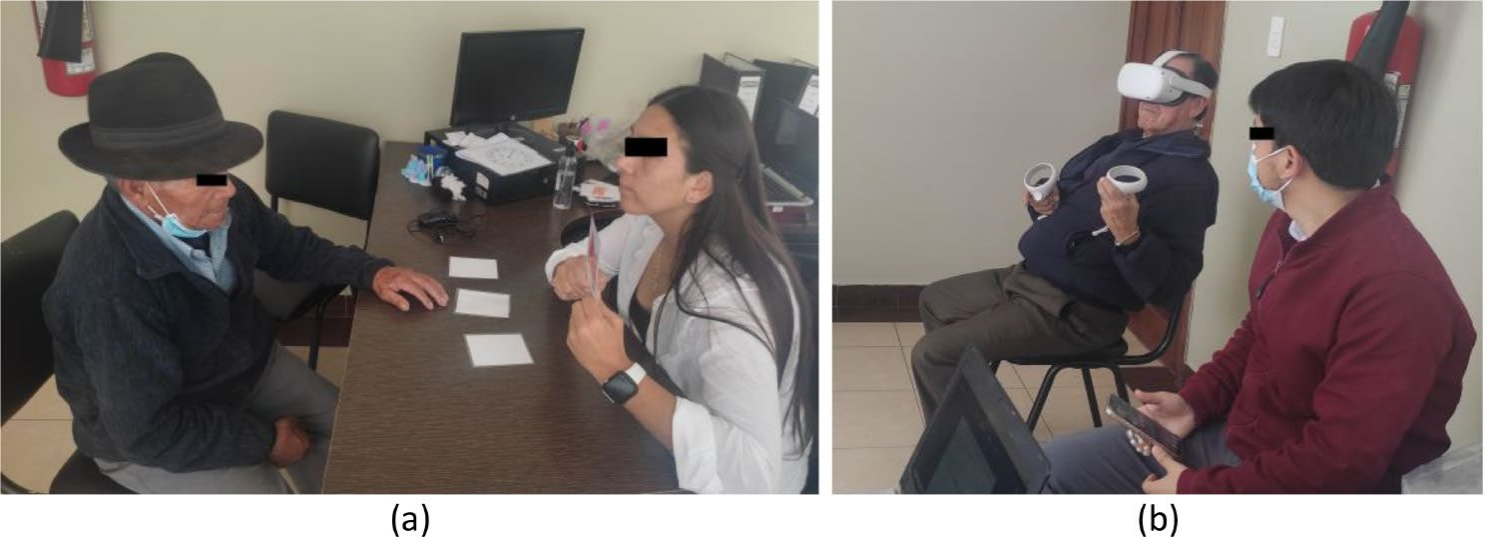
Participants performing the pencil and paper task **(a)** and the virtual task **(b)**

The intervention for the control group was performed under the same conditions as the experimental group maintaining the same sequential design, since the participants of this group also participated in the motor training through social activities, balance, resistance and low intensity aerobic exercises. After that, the individual cognitive training consisted of a task similar to that of the experimental group, but without the use of VR. Cards containing graphical representations of the same cooking ingredients as the virtual application were presented. A member of the research team was in charge of explaining the dynamics of the game to the participants and subsequently delivering the intervention. For 10 s the participant was allowed to memorize the picture of each of the cards and then the card was flipped over. In the recovery phase, the participant was asked to identify which card contained the requested ingredient and to turn it over to check that he/she got it right. Keeping the same instructions, the game started at level four and when the patient had no errors for two consecutive times, the level and therefore the number of elements to memorize increased. Considering that the reaction time could not be collected automatically as in the virtual application, another member of the team was in charge of taking these times with a stopwatch and recording them manually on a card. Like the experimental group, the participants played twice in each session, with the same weekly frequency and for the same total time period. Figure 4 shows some participants developing the task in both groups, together with members of the research team.

### Statistical analysis

Data were analyzed with SPSS version 24.0. Descriptive statistics, including mean, standard deviation (SD), were used to summarize the dependent variables within each group. Data normality was assessed with the Shapiro-Wilk’s test and homoscedasticity with Levene’s test. After checking the assumptions of normality and homoscedasticity, to evaluate the changes in cognitive function and depression status in older adults before and after the interventions, a paired t-test was performed to check within group differences whereas and independent sample t test was used to check for between groups differences. A one-way ANOVA with repeated measures was performed to check for differences in scores in each of the dependent variables at the two moments of time (pre and post intervention). The IADL-S scale data did not follow a normal distribution, so nonparametric tests were used to evaluate differences between measurements. The Wilcoxon signed-rank test (W) was used to analyze within-group changes over time, while the two-tailed Mann-Whitney (MW) test was used to compare differences between groups. As a complementary analysis, the Pearson’s correlation was used to analyze the degree of association between the MOCA-S and SGDS-S scores. The relationship between the level of education and the MOCA-S test scores will also be analyzed. The relationship between the level of education and the level achieved in the VR-based cognitive task will be also analyzed for the experimental group. The significance level was set at 0.05.

## Results

### Baseline data

The mean ages of the control group (*n* = 17) and the experimental group (*n* = 17) were 77.35 ± 6.75 years (range 65–84 years) and 75.41 ± 5.76 years (range 65–86 years), respectively. In the control group, the majority of the population was women (13/17), while in the experimental group the percentage of women was 58.82% (10/17). The level of education (measured in years) was on average 4.76 ± 3.25 years in the control group and 5.53 ± 3.18 years in the experimental group. In the control group, no one had previous experience using VR, while in the experimental group only one person had previously used VR. Two (11.8%) participants with arthritis were identified in both groups. In the experimental group, two (11.8%) people were observed with thyroid-related problems, prostate problems and diabetes. In the control group, high blood pressure was observed in five (29.4%) participants and in three (17.6%) of the experimental group. In summary, both groups were homogeneous with respect to gender, age, education, VR experience and presence of illnesses, respectively. Table 1 shows the demographic characteristics of the participants.

**Table 1:** Demographic characteristics of the groups

Variables	All (*N* = 34)	Control (*n* = 17)	Experimental (*n* = 17)	*P*-value
Gender, n (%)				0.271*
Male	11 (32.4)	4 (23.5)	7 (41.2)	
Female	23 (67.6)	13 (76.5)	10 (58.8)	
Age, n, SD (range)	76.38, 6.25 (65–86)	77.35, 6.75 (65–86)	75.41, 5.76 (66–84)	0.373
Education, n, SD (range)	5.15, 3.19 (0–12)	4.76, 3.25 (1–12)	5.53, 3.18 (0–12)	0.493
VR experience, n (%)				0.142*
Yes	1 (2.9)	0 (0)	1 (5.9)	
No	33 (97.1)	17 (100)	16 (94.1)	
Number of pre-existing illnesses, n (%)				0.76*
0	19 (55.9)	11 (64.7)	8 (47.1)	
1	10 (29.4)	4 (23.5)	6 (35.3)	
2 or more	5 (14.7)	2 (11.8)	3 (17.6)	

*Χ^2^ test

As far as the results at baseline in MoCA-S, SGDS-S and IADL-S, there were no significant differences between the groups in any of them (see Table 2).

**Table 2:** Neuropsychological evaluation tests score at baseline

Variables	All (*N* = 34)	Control (*n* = 17)	Experimental (*n* = 17)	*P* value
MoCA-S, n, SD (range)	21.74, 2.12 (19–27)	21.12, 1.73 (19–25)	22.35, 2.34 (19–27)	0.090
SGDS-S, n, SD (range)	5.00, 2.50 (1–10)	5.82, 2.16 (2–9)	4.18, 2.6 (1–10)	0.053
IADL-S, n, SD (range)	6.74, 1.58 (1–8)	6.47, 1.42 (3–8)	7.00, 1.73 (1–8)	0.12

MoCA-S: Spanish version of the Montreal Cognitive Assessment; SGDS-S: Spanish version of the Short Form of Geriatric Depression Scale; IADL-S: Spanish version of the Instrumental Activities of Daily Living Scale

### Post intervention results

Once the intervention was completed, the pre-posttest scores were analyzed and, depending on the distribution of the data, parametric or non-parametric tests were used. For the MoCA-S and SGDS-S tests (see Table 3), the paired samples t test was used, and the Wilcoxon test was used for the IADL scale (see Table 4).

**Fig. 5:**
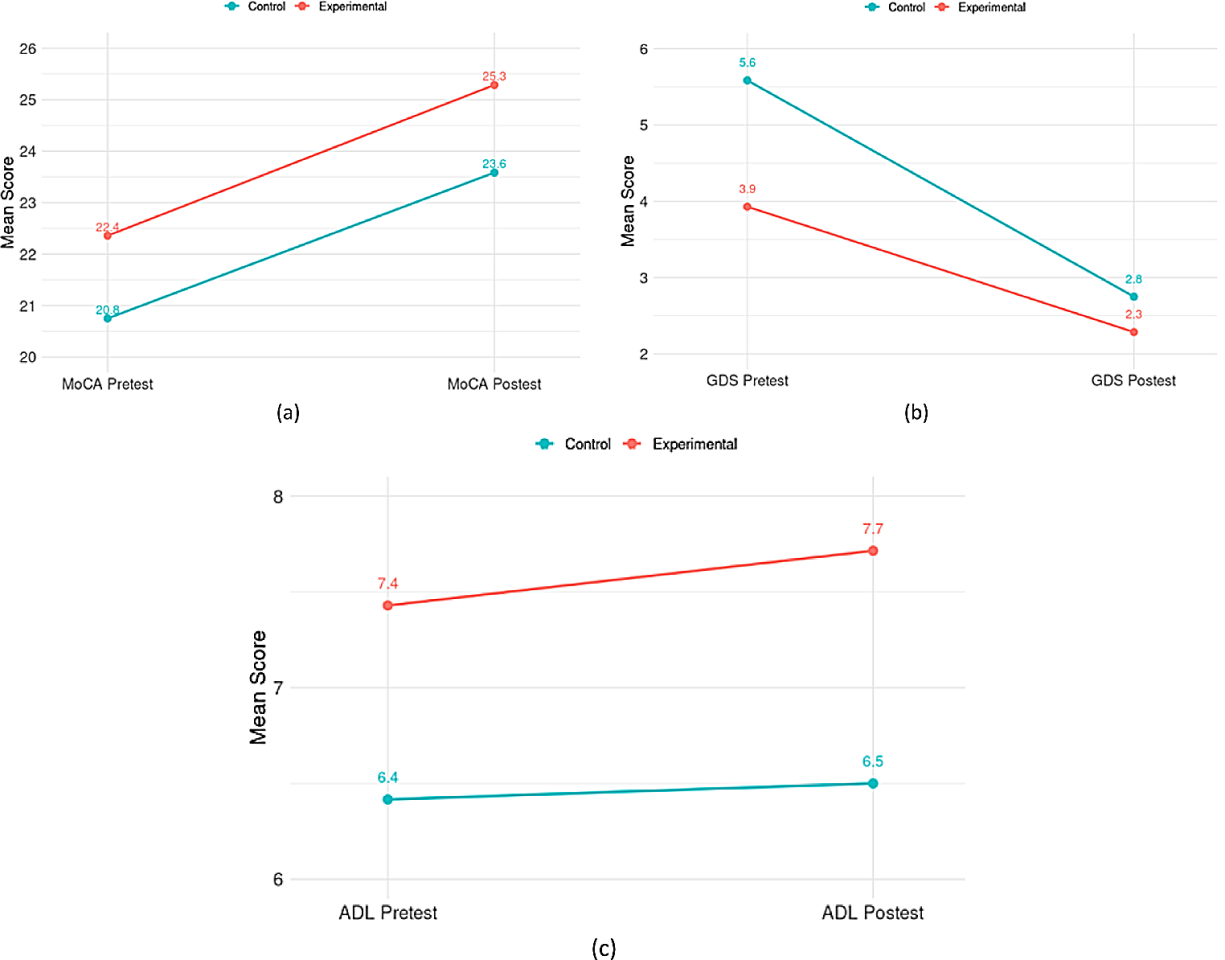
Pre- and post- intervention scores for control and experimental groups, respectively. **(a)** cognitive function; **(b)** geriatric depression; **(c)** performance of iADLs

The MoCA-S test scores showed an improvement in the cognitive functions of both groups, with a minimally greater effect in the control group (Fig. 5). The within group effect of cognitive function was significant in both groups (*p* < 0.001). When analyzing the SGDS-S scores, it was identified that both groups had a reduction in feelings related to depression, with a greater effect in the control group, showing a within group effect also significant. After intervention, the changes in the IADL-S test scores were slightly higher in relation to baseline in both groups (but not significant). Nevertheless, Mann-Whitney test revealed statistically significant differences between groups (in favor of the experimental group) (*p* = 0.002).

**Table 3:** Cognitive function and geriatric depression scores for both experimental and control group

Group, outcomes	Pre, mean (SD)	Post, mean (SD)	t value	*P* value	Cohen d
Control (*n* = 12)					
MoCA-S	20.83 (1.8)	23.67 (2.74)	-7.75	< 0.001	2.24
SGDS-S	5.58 (2.07)	2.75 (1.42)	7.34	< 0.001	2.11
**Experimental (** ***n*** ** = 14)**					
MoCA-S	22.36 (2.59)	25.29 (2.56)	-5.77	< 0.001	1.54
SGDS-S	3.93 (2.81)	2.29 (1.38)	3.37	0.005	0.90

MoCA-S: Spanish version of the Montreal Cognitive Assessment; SGDS-S: Spanish version of the Short Form of Geriatric Depression Scale

**Table 4:** Scores for iADLs performance for both experimental and control group

Group, outcomes	Pre, mean (SD)	Pre, median (min-max)	Post, mean (SD)	Post, median (min-max)	Z value	*P* value	Cohen d
Control (*n* = 12)							
IADL-S	6.42 (1.08)	6 (5–8)	6.50 (1.00)	6 (5–8)	-1.00	0.317	0.28
**Experimental (** ***n*** ** = 14)**							
IADL-S	7.43 (0.85)	8 (5–8)	7.71 (0.47)	8 (7–8)	-1.63	0.102	0.46

IADL-S: Spanish version of the Instrumental Activities of Daily Living Scale

### Task level of achievement

**Fig. 6:**
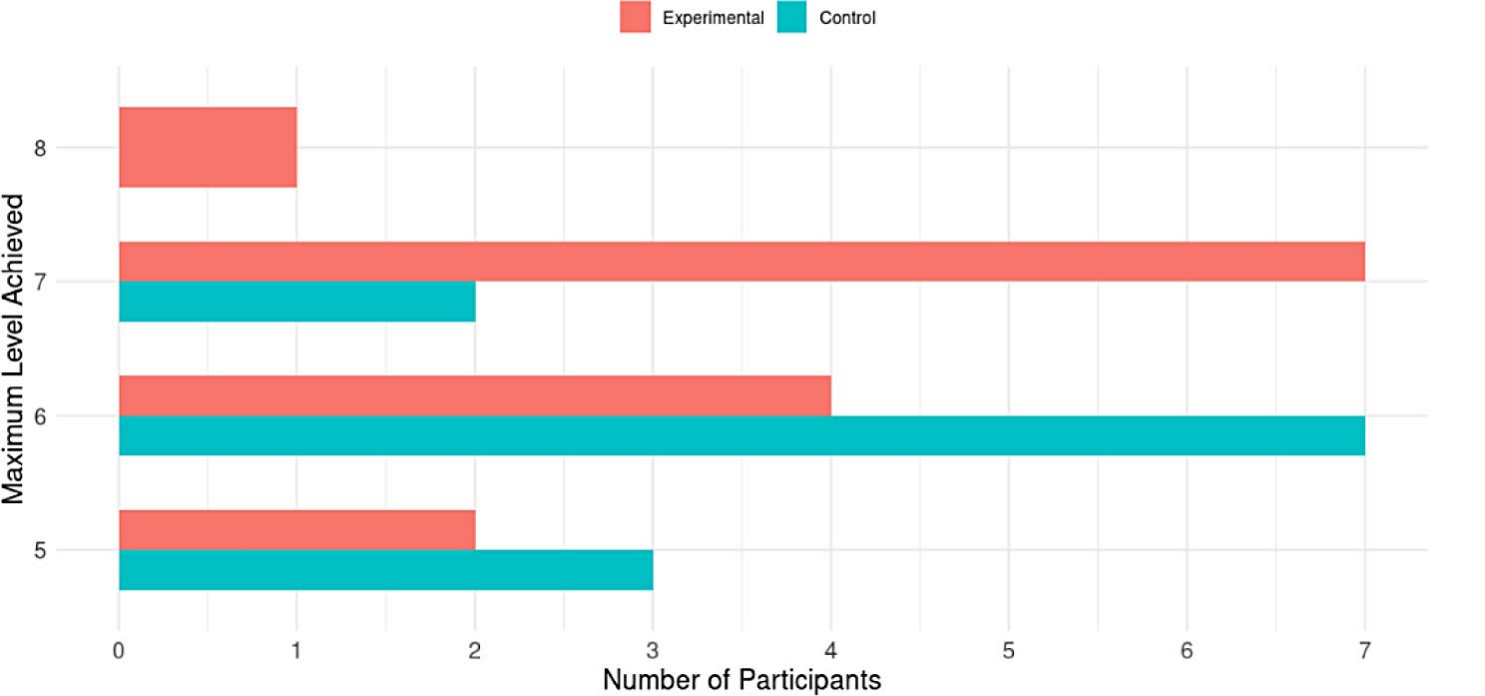
Distribution of the maximum level achieved by participants in the task in both groups

The intervention was completed by 70.59% (*n* = 12) in the control group and 82.35% (*n* = 14) in the experimental group, respectively. During the intervention period, each participant increased the level of difficulty within the task. At the end of the intervention, the maximum level reached by each participant was identified and these results were presented in Fig. 6. As can be seen, the majority (*n* = 7) of participants in the control group reached level 6 (memorizing up to 6 elements) and (*n* = 2) reached level 7. In the experimental group, the majority of the participants (*n* = 7) reached level 7 and even (*n* = 1) reached level 8.

**Fig. 7:**
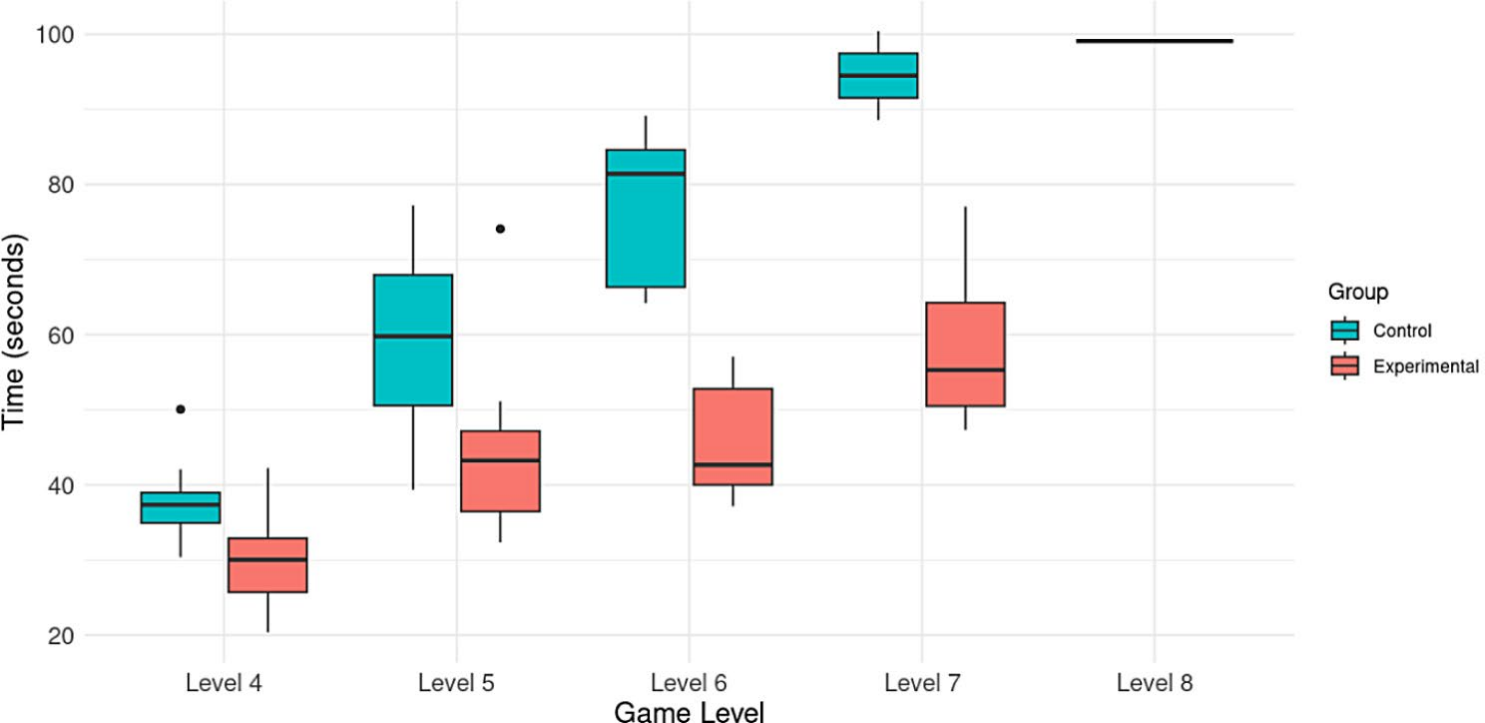
Average performance time categorized by level of difficulty and group

At the same time, the time it took each participant to complete the task in each of the sessions was analyzed. An average of the time used to perform the task by level was reckoned, whose results are presented in Fig. 7. As it can be seen, at level 4 the total completion time of the task was similar in both groups. The control group had an average time of 37.76 s and the experimental group had 30.37 s on average. As the level increases, increasingly significant differences can be seen in the average time of each group. At level 7, the control group spent 94.48 s on average, while the experimental group spent 58.88 s. The only participant who reached level 8 spent 99.07 s (on average).

### Gender differences

The MoCA-S test scores showed a statistically significant improvement in cognitive functions for both males and females in the experimental group (with very large effect sizes) and only for females in the control group (with an extra-large effect size). The SGDS-S scores showed a statistically significant reduction in depressive symptoms for females in both groups. The changes in the IADL-S test scores were slightly higher in relation to the baseline in both groups, but not significant. See Table 5 for details.

**Table 5:** Performance scores in cognitive functions, geriatric depression and iADLs performance in both groups classified by gender

Group, index	Male (*n* = 9)				Female (*n* = 17)			
	Pre, mean (SD)	Post, mean (SD)	*p*-value	Cohen d	Pre, mean (SD)	Post, mean (SD)	*p*-value	Cohen d
Control								
MoCA	20.33 (1.53)	23.33 (3.06)	0.095	1.73	21.00 (1.94)	23.78 (2.82)	< 0.001	2.31
SGDS	5.67 (3.06)	3.67 (2.08)	0.184	1.15	5.56 (1.88)	2.44 (1.13)	< 0.001	2.67
IADL	5.67 (0.58)	5.67 (0.58)	1.00	0	6.67 (1.12)	6.78 (0.97)	0.317	0.33
**Experimental**								
MoCA	21.67 (1.51)	25.5 (2.07)	0.005	1.97	22.88 (3.18)	25.13 (3.00)	0.007	1.35
SGDS	4.83 (3.49)	3.17 (1.6)	0.105	0.94	3.25 (2.19)	1.63 (0.74)	0.035	0.92
IADL	7.17 (1.17)	7.67 (0.52)	0.180	0.60	7.63 (0.52)	7.75 (0.46)	0.317	0.34

### Correlational analysis

Considering that the data of the MOCA-S and SGDS-S variables met the criteria of normality and homoscedasticity, a Pearson bivariate analysis was performed to examine the relationship between both variables. In the control group, analyses revealed a statistically significant association between scores obtained on the MOCA-S and SGDS-S after the intervention (Pearson *r* = -0.583, *p* = 0.047). This result could indicate that improvement or deterioration in cognitive function is associated with changes in depression levels. Additionally, level of education was found to correlate significantly with MOCA-S scores after the intervention (*r* = 0.706, *p* = 0.010). In the experimental group, there was no association between level of education and the maximum level achieved in the virtual task (*r* = 0.492, *p* = 0.074), suggesting that education does not have a positive impact on the cognitive task performance. By contrast, there was a statistically significant association between level of education and MOCA-S scores before (*r* = 0.610, *p* = 0.009) and after the intervention (*r* = 0.799, *p* = 0.001).

## Discussion

The increasing incidence of AD emphasizes the need to address its complexity, caused by the interaction of genetic and environmental factors [48]. Given the limited effectiveness of pharmacological treatments in their initial phases or in their prevention, the importance of exploring non-pharmacological alternatives arises [51]. Our study presented a behavioral intervention that, unlike pharmacological interventions, has broader eligibility criteria, which contributes to the generalization of results. This approach aligns with previous research conducted in Europe and Asia demonstrating that VR could be used as a preventive measure against dementia [47, 48].

This study was carried out in a Latin American population, seeking to contrast its findings with previous research in contexts where the use of virtual environments could be more common. We remark the good adherence and successful training in the sample. This approach also appears to be safe, as participants reported no adverse effects during the intervention. Preliminary results indicate that this program could be an effective tool to improve cognitive function in older adults with MCI. It is also suggested that there is efficacy in reducing depressive symptoms in this population. However, the results do not show a direct transfer of skills to everyday life situations, pointing out the need for more research to explore this aspect.

### Effects on cognitive functions

Our study has shown that a dual intervention (sequential motor and cognitive training) can generate significant improvements in people with MCI. MoCA-S test scores reflected significant improvements in the cognitive functions of both groups. The control group showed notable progress, increasing its average score from 20.83 to 23.67, approaching the threshold of cognitive normality. Although there are studies such as that of Manser et al. [73], who mention that virtual environments could be a limitation due to the difficulty of using the system, our study showed promising results. The experimental group, which received the cognitive training via VR technology, increased MoCA-S test scores from 22.36 to 25.29, very close to normal cognitive performance. These improvements, in addition to being clinically significant, highlight the potential of VR-assisted cognitive rehabilitation.

The methodology used improved specific cognitive functions, including executive function, spatial navigation and memory. Our study was based on recent literature indicating that combined cognitive-motor training can exceed the effectiveness of isolated cognitive or motor training, as demonstrated by notable improvements in cognitive performance metrics [46, 74]. Doniger et al. [51] highlighted that cognitive and physical training exert distinct but complementary neurobiological benefits. Cognitive training has been shown to increase executive function by increasing cerebral blood flow (CBF) in the prefrontal cortex and medial/posterior cingulate cortex. On the contrary, physical training has been associated with improved memory, correlating with an increase in CBF in the hippocampus. These findings corroborate the premise that each training modality uniquely contributes to neurorehabilitation processes and promotes neuroplasticity, thereby facilitating brain repair and functional recovery [75].

The ability to strengthen cognitive reserve and stimulate neuroplasticity makes individuals with MCI ideal participants for this study [76]. Despite the neural declines associated with aging, different models have proposed explanations for how the brain can maintain optimal cognitive functions. One of these, the compensatory model, indicates that the brain achieves this through increased activation of certain specialized areas [77]. In this sense, virtual environments provide constant visual and spatial stimuli, via HMD, achieving interaction with real-time visualization. This not only increases CBF in the previously mentioned areas, but could also explain the improvement in neural efficiency observed in [78], suggesting better post-training cognitive performance. This impact has the potential to strengthen cognitive abilities and slow the progression of cognitive decline in older people at risk of developing dementia [48].

To achieve the expected results in any intervention, adherence to the training program is very important. In our intervention, there was a higher completion rate of participants in the experimental group, which shows the relevance of motivation for adherence to the program. This observation is consistent with the results of a meta-analysis highlighting the importance of motivation to increase active participation in this type of program [79]. The inclusion of VR technologies, by providing immediate feedback and generating a stimulating training environment, can overcome the demotivation frequently observed in cognitively impaired older adults [80]. It is argued that the increased engagement, motivated by the interest and enjoyment elicited by VR, activates neural mechanisms that promote concentration and memory through stimulation of neurotransmitter systems, such as cholinergic and dopaminergic systems [81]. It can also be added that using VR to simulate an everyday environment could be more effective in increasing motivation and thus achieving significant cognitive improvements, which is consistent with our study [52]. In contrast, traditional physical and cognitive training programs do not usually offer these motivational aspects.

In our research, we observed a statistically significant and positive relationship between the level of education of the participants and their MoCA-S scores, suggesting that a higher level of education is associated with better cognitive performance [82]. It is worth noting that, within our sample, only two participants completed their high school education, while the majority did not complete their basic education. However, they possess basic reading and numeracy skills. This observation aligns with the findings of Man et al. [83], who reported that individuals with lower educational level and mild dementia could derive greater benefits from training focused on ADL. Complementarily, Mondini et al. [84] concluded that those with lower cognitive reserve may be particularly susceptible to improvement through cognitive rehabilitation. Future research could focus on assessing the influence of additional factors, such as socioeconomic, cultural, linguistic context or disabilities.

### Effects in depression

Depression in the geriatric population is a condition that can negatively affect cognitive function and quality of life. Although the main focus of the study was the improvement of cognitive functions, depressive symptoms are related and our results could provide relevant information [55]. The results of this study are encouraging, as they indicate that both traditional and VR-assisted interventions may be useful in reducing depressive symptoms. Initially, the control group was in the range of mild depression and, after the intervention, it was in a normal state (GSDS test from 5.58 to 2.75). While the experimental group had a reduction from 3.93 to 2.29, which, although it was already within the normal range, now presents even more reduced depressive symptoms. Both results are statistically significant (*p* < 0.001 for the control and *p* = 0.005 for the experimental), indicating that both intervention modalities were effective in reducing depression, although the improvement was slightly more pronounced in the control group.

Despite the benefits observed with the use of VR that highlight its potential as a complementary tool, the most significant results in terms of mental health were recorded in the control group. One interpretation of this phenomenon suggests that the pencil and paper task promoted a closer interaction between the therapist and the participant, favoring a communicative bond through conversations during breaks, aspects not contemplated in the original design of the study, but inherent to the therapeutic process. This agrees with [85] who mentions that cognitive training based on card games and accompanied by social interaction improves executive control in older adults. In the study of Langoni et al. [86], group exercises improved balance, mobility, and depressive symptoms in community-dwelling older adults with MCI. This finding highlights the positive impact of social integration and family support on the well-being of older adults, highlighting the importance of considering the environment in the design of the intervention.

The methodology of this study was designed to overcome the limitations of previous research, where the use of an inactive or low-contact control group has been criticized [87]. Previous studies, such as those carried out by Brito et al. [64] and Trueba et al. [65], did not incorporate active controls, which limits the interpretation of their findings. This omission restricts the ability to determine the genuine impact of their interventions. Our research chose to compare the use of VR with a control group that participated in active cognitive training accompanied by social interaction exercises. Additionally, analysis of the data by gender revealed that women in both groups showed a greater reduction in depressive symptoms compared to men. This finding is consistent with recent studies, such as that of Kim et al. [88], which showed the significant potential of VR applications to positively impact the female population.

The initial inclusion of motor, balance, and aerobic exercises not only promoted social interaction between participants and therapists in a real-world environment, but also established a foundation for effective social interaction [89, 90]. The use of VR in cognitive training added a unique dimension by providing visual and auditory feedback that recognized participants’ achievements, fostering a sense of accomplishment and mitigating loneliness. This tool was especially valuable for those participants who, due to their advanced age or limitations, had distanced themselves from everyday activities such as food preparation, allowing them to interact in a virtual environment that simulated these tasks. This is relevant in developing countries, where, according to the meta-analysis by Zenebe et al. [32], the prevalence is almost three times higher compared to developed countries.

Another factor that could explain the difference in outcomes deals with the choice of an immersive system. A previous systematic review notes that older adults experience more positive effects when using commercial gaming systems such as Nintendo Switch or Xbox Kinect. This is attributed to the level of interaction, entertainment, and fun they offer, making them more engaging and potentially more effective in reducing depressive symptoms [55].

### Effects in IADLs

A recent review highlighted how virtual environments that simulate iADLs could have greater ecological validity and foster autonomy in daily life [91]. The importance of these applications lies in their complexity and relevance, motivating participants to plan, organize, solve problems and manage multiple tasks simultaneously within a spatial and visual context [51]. This approach not only improves specific skills, but could ensure that learning is directly applicable to real situations, thus facilitating greater independence in daily activities [92]. Our intervention, based on the cupboard task, differs from applications that simulate more traditional IADL such as preparing food or shopping. Participants had to remember objects, for which they planned future actions, but the locations changed in each experiment to avoid monotony in the game. This approach trained participants in updating and managing dynamic information in their working memory, a relevant component of prospective memory, supporting cognitive flexibility and executive functions [93].

Despite the ecological validity of VR and the theoretical basis for real-life skill transfer, results did not demonstrate significant improvement on subsequent IADL tests, which can be interpreted as a lack of improvement in functional independence. This finding is consistent with the study of McDaniel et al. [87], who suggest a limit on the potential for improvement, due to a possible functional ceiling among some participants, who already have a high level of performance and, therefore, have less room to show significant improvements. On the other hand, some elderly people are under the care of relatives so they have lost autonomy to carry out tasks such as shopping or preparing food [94]. Fjordside and Morville [95] highlighted that older people have internal motivation to preserve their independence within their homes. This independence is compromised as the person becomes more dependent on the assistance of their caregivers. Therefore, people institutionalized in a geriatric center did not participate. The perception of autonomy of older people is closely related to their social relationships, the care practices of their caregivers and the residential environment, respecting their needs [96]. This context suggests that improving these skills in a short period may not be feasible.

Furthermore, Lawton and Brody’s IADL scale may not have been sensitive enough to capture significant changes in participants’ daily lives, given that the study was not designed to induce changes of such magnitude [97]. However, a specific improvement in smartphone use was observed, indicating an increase in comfort and motivation towards the use of smart devices among participants in the experimental group. The recent study of Wei and Guo [98] found that smartphone use had a positive effect on the health status of older adults. This specific result suggests that, although the intervention was not able to broadly improve IADL skills, it was able to positively influence specific aspects of interaction with technology.

### Limitations

A systematic review suggested that to achieve maximum therapeutic efficacy, interventions should last at least six weeks [55]. Another meta-analysis stated that interventions should have a minimum of 10 sessions to facilitate transfer of learning [99]. Although our study complies with these conditions, we consider that a possible limitation was its duration and that a longer period would have allowed for greater consistency in the results. Our sample size is consistent with previous studies, but is still small, which limits confidence in the observed effects.

Another limitation is related to the lack of a follow-up evaluation, which was considered within the initial study to be carried out four months later. However, some participants stopped attending the senior community center and others did not wish to participate in the evaluations, which made this process impossible. In addition, the study was conducted exclusively in a senior center, thus limiting the generalizability of the results. The question arises as to whether the observed effects would be consistent in populations that do not frequent these centers, suggesting the need to expand the scope of future research to include diverse settings and populations.

An important aspect that should be controlled in future studies is the effect that social interaction can have on final performance. In our intervention, greater social interaction was revealed by the control group, which could have favored this group and justified the results obtained.

## Conclusions

This study presented a VR-based intervention that obtained significant improvements in executive function, navigation and memory, as well as in the reduction of depressive symptoms. The results were comparable to those obtained with a traditional intervention that was provided by a therapist, which opens the door to the use of VR-based cognitive therapies in community-dwelling older adults provided by family members or caregivers, and without the permanent need of a therapist. The high rate of adherence to the program in the experimental group highlights the relevance of designing motivating and attractive VR interventions.

Future research should address the limitations of this study, such as its duration and sample size, to consolidate the current findings. Additionally, long-term follow-up evaluations should be conducted, as well as expand the scope of the research to a broader variety of contexts and populations. Investigating the effectiveness of VR in improving depressive symptoms and transferring cognitive skills to daily activities is promising field, which requires further exploration to maximize the therapeutic potential of this technology in healthy aging.

## Data Availability

No datasets were generated or analysed during the current study.
