# Correction: Effects of a dual intervention (motor and virtual reality-based cognitive) on cognition in patients with mild cognitive impairment: a single-blind, randomized controlled trial

**DOI:** 10.1186/s12984-024-01443-5

**Published:** 2024-08-31

**Authors:** Jorge Buele, Fátima Avilés-Castillo, Carolina Del-Valle-Soto, José Varela-Aldás, Guillermo Palacios-Navarro

**Affiliations:** 1 Carrera de Ingeniería en Tecnologías de la Información, Facultad de Ingeniería, Industria y Producción, Universidad Indoamérica, Ambato, 180103 Ecuador; 2 Centro de Investigaciones de Ciencias Humanas y de la Educación (CICHE), Universidad Indoamérica, Ambato, 180103 Ecuador; 3 Facultad de Ingeniería, Universidad Panamericana, Álvaro del Portillo 49, 45010 Zapopan, Jalisco Mexico; 4 Department of Electronic Engineering and Communications, University of Zaragoza, Teruel, Spain; 5 Teruel Polytechnic School of Engineering, University of Zaragoza C/Atarazana, 2, 44002 Teruel, Spain

**Corection: Journal of NeuroEngineering and Rehabilitation (2024) 21:130** 10.1186/s12984-024-01422-w

Following publication of the original article [1], the author’s name ‘Carolina Del-Valle-Soto’ was incorrectly written as ‘Carolina de Valle Soto’.

The original article has been corrected.
